# An integrated community mental healthcare program to reduce suicidal ideation and improve maternal mental health during the postnatal period: the findings from the Nagano trial

**DOI:** 10.1186/s12888-020-02765-z

**Published:** 2020-07-29

**Authors:** Yoshiyuki Tachibana, Noriaki Koizumi, Masashi Mikami, Kana Shikada, Sayaka Yamashita, Mieko Shimizu, Kazuyo Machida, Hiroto Ito

**Affiliations:** 1 Division of Infant and Toddler Mental Health, Department of Psychosocial Medicine, National Center for Child Health and Development, 2-10-1 Okura, Setagaya-ku, Tokyo, 157-8535 Japan; 2 Nagano Prefectural Center for Mental Health and Welfare, Nagano, Japan; 3 Department of Biostatistics, Clinical Research Center, National Center for Child Health and Development, Tokyo, Japan; 4 Nagano City Public Health Center, Nagano, Japan; 5 Japan Organization of Occupational Health and Safety, Kanagawa, Japan

**Keywords:** Suicide ideation, Edinburgh postnatal depression scale, Maternal mental health, Postnatal care

## Abstract

**Background:**

During the perinatal period, suicides are more likely to occur in those with depression and who are not receiving active treatment at the time of death. Suicide is a common outcome in people with suicide ideation. We developed an intervention program taking care of comprehensive perinatal maternal mental healthcare to prevent suicide ideation. We hypothesized that our intervention program could reduce postnatal suicide ideation and improve maternal mental health.

**Methods:**

We performed a controlled trial to examine the usual postnatal care plus a maternal suicide prevention program (the intervention group) compared with usual postnatal care alone, which comprised home visits by public health nurses without mental health screening (the control group) in Nagano city, Japan. In total, 464 women were included; 230 were allocated to the control group and 234 to the intervention group. The intervention had three components: 1) all the women received postnatal mental health screening by public health nurses who completed home visits during the neonatal period, 2) the intervention was administered by a multidisciplinary clinical network, and 3) systematic follow-up sheets were used to better understand bio–psycho–social characteristics of both the mothers and their infants and develop responsive care plans. We measured the participants’ mental health at 3–4 months postpartum (T1) and 7–8 months postpartum (T2) using the Japanese version of the Edinburgh Postnatal Depression Scale (EPDS).

**Results:**

Suicidal ideation was significantly lower in the intervention group compared with the control group at T1 (*p* = 0.014); however, this significant between-group difference did not continue to T2 (*p* = 0.111). We measured the intervention effects on maternal mental health using the total score of the EPDS, which was significantly improved in the intervention group compared with the control group at T1. Here, the significant difference continued to T2 (*p* = 0.049).

**Conclusions:**

Our results indicate that our program may reduce maternal suicidal ideation at 3–4 months postnatally and improve women’s mental health during the postnatal periods of 3–4 to 7–8 months. Postnatal maternal mental healthcare, including services to reduce suicide ideation, should be included as an important component of general postnatal care.

**Trial registration:**

Name of registry: A multidisciplinary intervention program for maternal mental health in perinatal periods.

UMIN Clinical Trials Registry number: UMIN000033396.

Registration URL: https://upload.umin.ac.jp/cgibin/ctr/ctr_view_reg.cgi?recptno=R000038076

Registration date: July 15, 2018.

Registration timing: retrospective.

## Background

Suicide is a principal cause of perinatal maternal deaths in developed countries [1, 2], and it commonly occurs following a period of suicide ideation [3]. Thus, suicidal ideation can be a pivotal target for preventing suicide, and many preventive interventions exist that seek to reduce suicidal ideation [4–7].

In the perinatal period, about one out of ten perinatal women exhibit suicidal ideation women exhibit suicidal ideation; Howard et al. reported that 9% of mothers at 6 weeks postpartum had suicidal ideation, determined via the Edinburgh Postnatal Depression Scale (EPDS) [8], and 4% of the women had frequent suicidal ideation [9]. Mental healthcare interventions for use during the perinatal period have been developed, and target various psychological and psychosocial problems (e.g., depression [10, 11], anxiety [12, 13], and mother–infant attachment [14, 15]).

However, intervention programs in the perinatal period that target suicidal ideation have not been performed, although some clinical practice guidelines mention the need to manage suicide ideation [16]. Hence, programs for reducing suicidal ideation, potentially preventing maternal suicides during perinatal period, are needed. Khalifeh et al. reported that suicides during the perinatal period were more likely to occur in those with depression who were not engaged in active treatment at the time of death [17]. They suggested that assertive follow-up and treatment of women in contact with psychiatric services during the perinatal period were important for preventing suicides among at-risk new mothers. Developing an intervention program that includes both treatment and follow-up is needed, and such a program could be integrated into existing maternal and child healthcare practices.

Various types of effective approaches have been developed for suicide prevention in non-perinatal areas, e.g. school-based programs [18–22], public awareness campaigns [23–26], primary care physician education [27], gatekeeper training [28–31], media reporting [32–35], media blackout [36], and screening [37–42]. These suicide prevention methods can be classified as population approaches (e.g. public awareness campaign, media reporting, media blackout) or high-risk approaches (e.g. school-based programs, primary care physician education, gatekeeper training, screening).

When weaving suicidal ideation prevention programs into existing maternal and child health services, certain characteristic points of suicide prevention programs in the perinatal period compared to those in the non-perinatal period should also be considered. One of these characteristics is that the perinatal period has some optimal time points when intervention would be most useful e.g. at the times of pregnancy health examinations and home visits for newborns.

In Japan, public health nurses perform home visits for all mothers and their babies within 4 months after birth. These public health nurses usually perform a mental health screening (EPDS) and psychosocial risk assessments at the newborn home visit for all postpartum mothers, which can be regarded as a population approach. If the nurses notice any psychosocial risks for these mothers and their babies, they can then enact care for the family intensively. This process, by contrast, is a high-risk approach. Thus, to develop an intervention program in the perinatal period, both population and high-risk approaches should be considered for characteristic service systems in the perinatal period.

Another important characteristic of suicide prevention programs in the perinatal period compared those in the non-perinatal period is that a large number of related professionals can be involved, including public health nurses, midwives, nurses, obstetricians, pediatricians, psychiatrists, medical social workers, and child welfare social workers, compared to other intervention programs. Since there are many professionals involved, performing collaborative support for mothers and children can sometimes be complex and difficult [43]. A previous study suggested that multi-professional meetings for supporting mothers and children at risk of psychosocial problems facilitated the collaboration among such a large number of professionals [44]. Furthermore, the multi-professional support system was shown to improve the mental health among postpartum women.

Since postnatal health services differ among countries, it is important to consider country-specific aspects of standardized care. The World Health Organization suggested a strategy that promotes standardized antenatal care, skilled birth attendance and early postnatal care. In their promotion, they suggested that newborn and maternal care during home visits be performed in compliance with the approaches of country and according to local health service resources [45]. They also suggested the importance of the “continuum of care” which has two meanings: the continuum of the lifecycle from adolescence to pre-pregnancy, pregnancy, birth and the newborn period, and the continuum of care from the home and community to the health center and hospital and back again. In Japan, two national home visit programs have been enacted. One is the ‘newborn home visit project’, which instructs new parents on the important points to bear in mind for their newborn (e.g. development, nutrition, life environment, disease prevention), provides care to needy mothers and newborns and supports mothers within 28 days postpartum (or for mothers living with either their parents or parents in law, within 60 days postpartum). The other program is ‘the Home Visit Project for All Infants’, which provides counselling on parenting, helps support parenting efforts, clarifies the newborns and their parents’ conditions as well as the child-rearing environment and evaluates and helps draft support plans for families in need.

Both of these home visits are performed by public health nurses and midwives. These systems have made it possible for Japanese health services to deliver postnatal and newborn care to postpartum women and their newborns as a population approach. Adding a suicidal risk assessment and care to these pre-existing postnatal care systems can aid in the implementation of suicide prevention programs as a population approach in the postnatal period.

We propose a clinical management intervention program for preventing suicide in postnatal women at risk of psychosocial problems, using assertive treatment, contact with psychiatric services, and follow-up as needed. The program focuses on reducing suicidal ideation in postnatal women at risk of psychosocial problems and improving their mental health. We developed a clear referral and management pathway for antenatal and postnatal women with psychosocial problems [44–47]. The present study’s intervention functions in combination with our earlier mother and child healthcare support program that spans the various pregnancy periods. The intervention involved mental health screening to detect women with suicidal ideation or who were at risk of psychosocial problems. The intervention also involved a clear referral and management pathway for managing those women. We hypothesized that the intervention program could reduce suicidal ideation and improve maternal mental health. To examine this hypothesis, we performed a controlled study to investigate program effectiveness.

## Methods

### Study design and participants

We selected Nagano city as our research field because of its rural areas, urban areas, and industrial areas, which reflect a range of other areas in Japan. Nagano is the capital city of the Nagano prefecture, and its population was 374,196 in 2015 and 372,175 in 2016.

The intervention program was performed as a component of Nagano city’s and Nagano Prefectural Mental Health and Welfare Center’s maternal and child care health services. The study protocol was reviewed and approved by the Institutional Review Board of the National Center for Child Health and Development, Japan. All women provided their written informed consent to participate in this historical controlled trial to compare usual care, plus maternal suicide prevention program, to the usual care alone. Here, usual care consisted of home visits by public health nurses, without mental health screening. All of the 17 public health centers in Nagano city, and their public health nurses, were involved in this study, which was supported by the Japanese Ministry of Health, Labor and Welfare. The sole inclusion criterion was submission of a birth notification to the Nagano city government. There were no exclusion criteria for this study. Informed consent was obtained upon submission of a birth notification form to the local government.

**Fig. 1:**
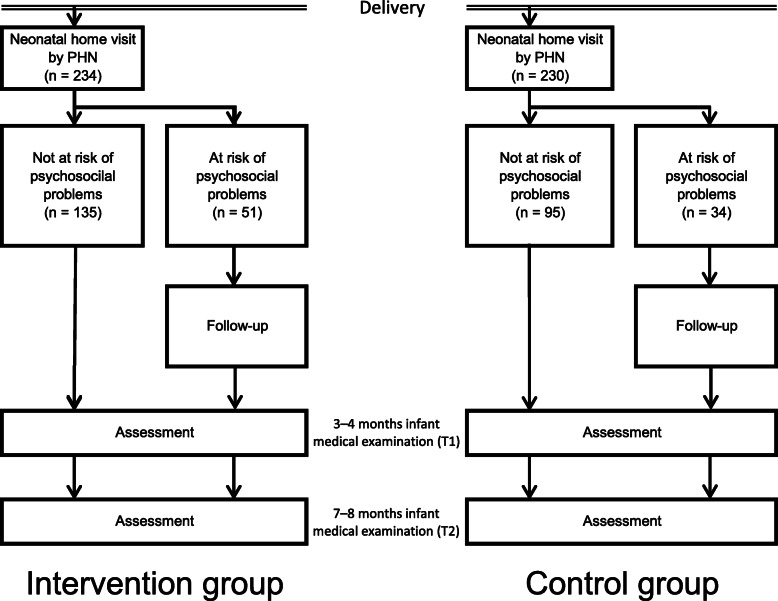
Flow chart of the current study

This study included 464 women, 230 of which had submitted their birth notification forms to the Nagano city’s local government from November 2015 to March 2016 were allocated into the control group. The remaining 234 women who had submitted their forms between April 2016 and July 2016 were allocated to the intervention group. Those who were identified at risk of psychosocial problems were followed up by the public health nurses (Fig. 1).

### Interventions

Interventions were designed on the basis of our previous intervention program (the Suzaka trial) [44], preliminary surveys [48–50] clinical guidelines for perinatal mental health [51], and discussions among the researchers and with healthcare professionals in the study regions, with a focus on preventing postnatal suicide. The intervention had three characteristics as below based on our previous intervention program [44]. Firstly, the multidisciplinary maternal and child health service team including public health nurses, obstetricians, psychiatrists, midwives, nurses, medical social workers, and pediatricians shared the referral and management protocol (Additional files 2 and 3). Secondly, the multidisciplinally maternal and child health team in case the public health nurses or the other professionals related mother and child noticed psychosocial risks with the family, the mulitidisciprinally maternal and child health service team follow up them in corporation with each other (high risk approach). In addition to those Suzaka trial’s intervention system, the present study’s intervention program had a characteristic approach using case management interventions for women with suicidal ideations.

All the postnatal women received a mental health screening with the EPDS by the public health nurses who did home visit in the neonatal periods. The public health nurses administered the self-harm question of the EPDS, which is a question related to suicide ideation [9], also noting any past psychiatric history of schizophrenia, major depression, or bipolar disorder [46–48]. In instances where the public health nurse identified a mother as exhibiting suicide ideation, the TALK steps (i.e., Tell, Ask, Listen, Keep safe) were performed for their psychological crisis interventions for that visit [52], and all follow-ups. The public health nurses assessed psychosocial risk comprehensively including suicidal risk. Based on the results of the assessments, the public health nurses performed psychoeducation for the women, and their family if it was possible. Psychoeducation is effective to prevent suicide [53]. The public health nurses performed the following psychoeducation. First, the public health nurses taught them the risk of narrow-mindedness when they have suicidal ideation, and in such situations, that they tend to be isolated with sense of hopelessness. The public health nurses also taught them the necessity of psychological or psychiatric cares to relieve their suicidal ideations. They also taught them that medications can also be effective. The public health nurses also inquired about family support conditions, and collaborated with family members to generate support for the mothers, if possible. They urged the mothers to promise not to harm themselves or their children, and agreed upon a “SOS” signal that could be directed toward specialists such as psychiatrists or public health nurses in case of a mental health crisis. In cases that the women’s families were concerned with or exhausted with their cares, the public health nurses also heard attentively and empathically, they respect their families’ opinions and think together how they should manage the problem.

**Fig. 2:**
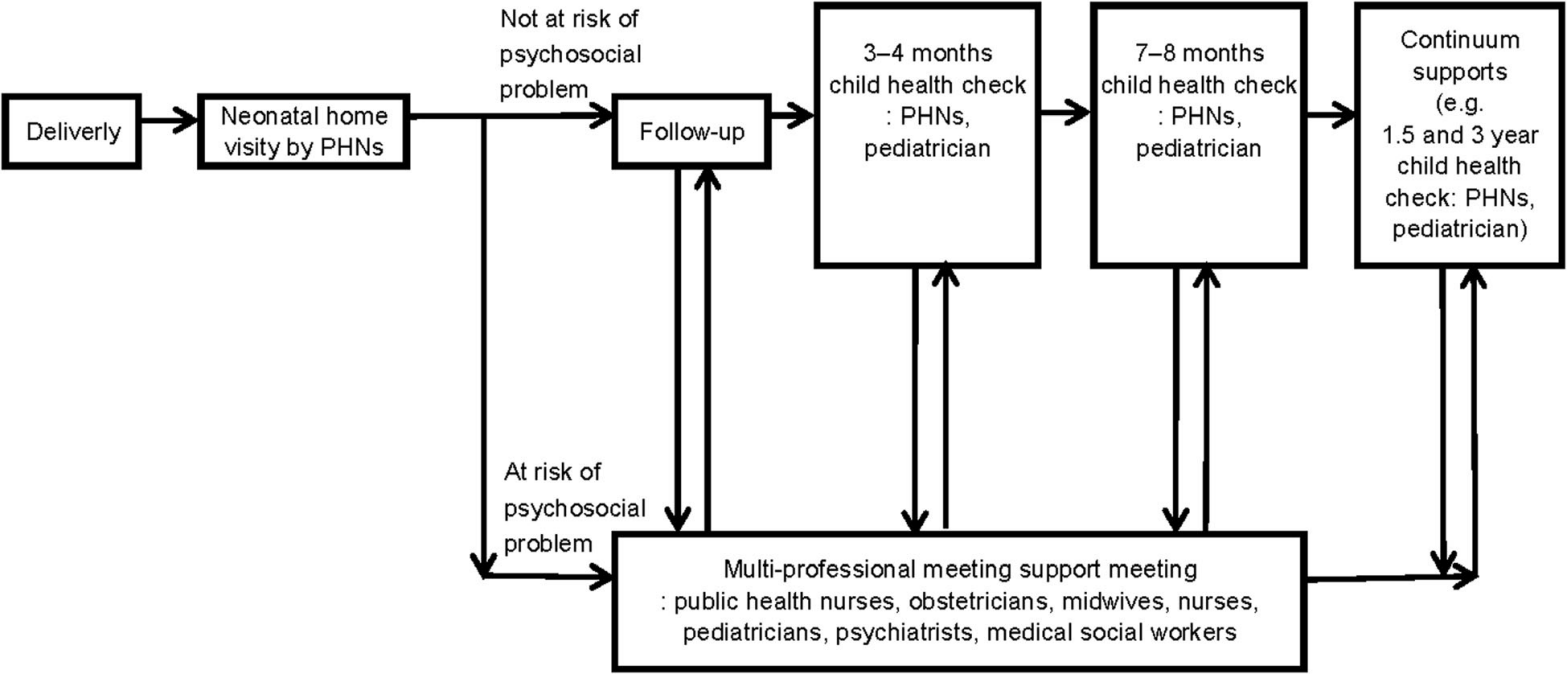
Shema of Nagano trial’s continuum and multidisciplinary maternal and child health service. Footnotes: PHN - public health nurse; EPDS - Edinburgh Postnatal Depression Scale

The intervention program was performed by a multidisciplinary clinical network, in consideration of the recommendations for perinatal mental health services as per National Institute for Health and Care Excellence (NICE) guidelines [16]. The NICE guideline suggested the models of managed clinical networks with reference to Goodwin and colleagues” “the characteristics of successful networks” [54]. We referred the models for developing the present study’ program. The clinical network involved mother and child health service hospitals and institutes in Nagano city, and consisted of multidisciplinary perinatal service professionals including gynecologists, midwives, nurses, public health nurses (who did the home visits), pediatricians, social workers, and psychiatrists (Fig. 2), under the support of the Nagano city public health center and the Nagano City Medical Association.

**Fig. 3:**
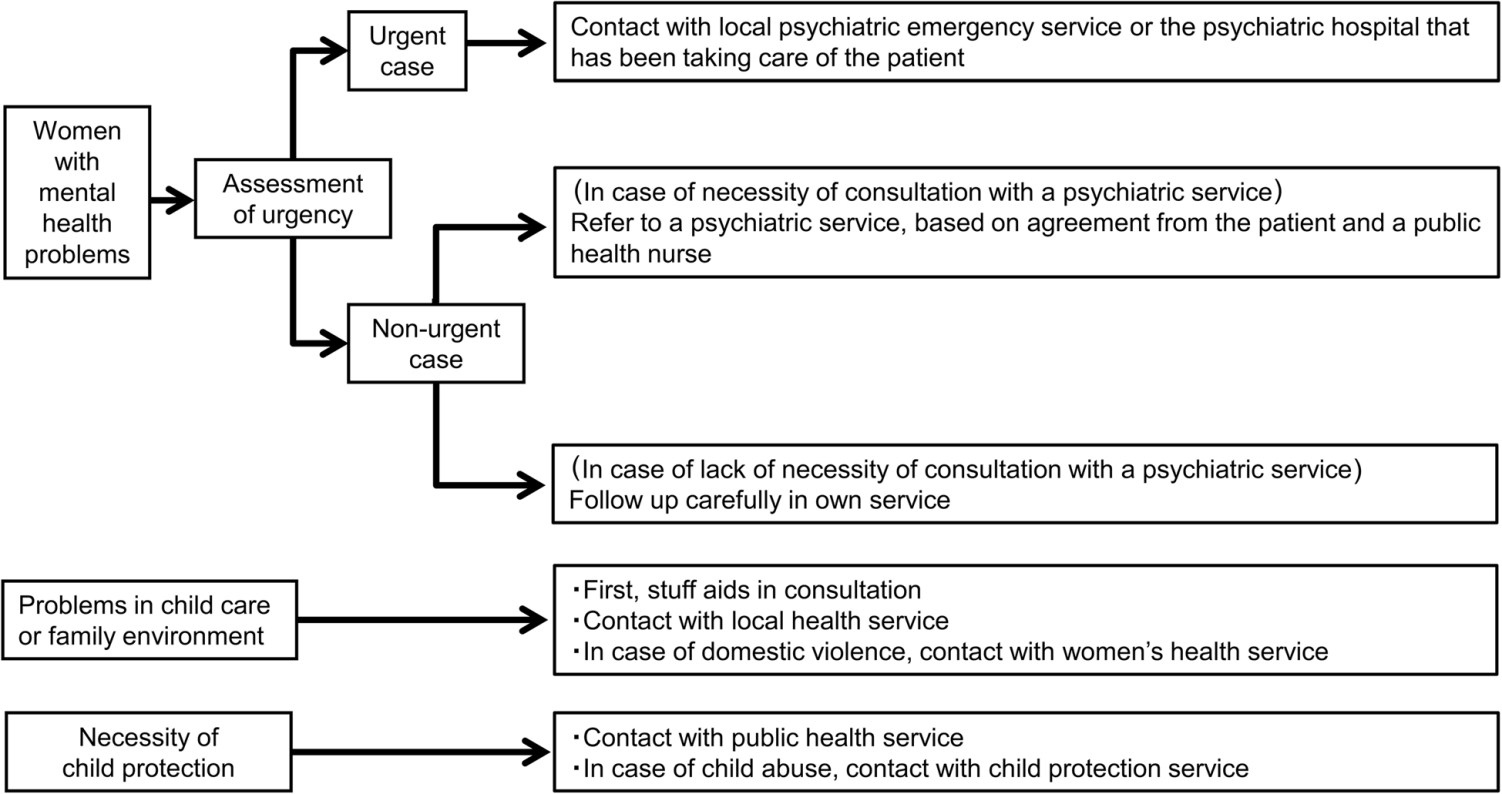
Referral and management protocols for services for women and children with psychosocial problems. Footnotes: For a woman with a past history, or familial history, of a severe psychiatric disorder such as major depressive and bipolar disorder, the occurrence of psychiatric symptoms was assessed, and the women were referred to psychiatric services if necessary. “Urgent case” indicated i) strong suicidal ideas or intention of self-harm that could not be self-managed; ii) sudden emergence of psychotic symptoms; iii) risk of self-harm or causing harm to others

The part-time psychiatrists of the public health centers provided expert advice on the risks and benefits of psychotropic medication use during pregnancy and breastfeeding for the mothers with whom they consulted. Related mother and child health service professionals in Nagano city followed the referral and management protocols for services across all levels of the existing stepped-care frameworks [55] for maternal mental health problems, problems related to child care or the family environment, and child protection. Nagano city has referral and management pathways for mothers and children beginning in pregnancy (Fig. 3).

This protocol pays particular attention to assessments of urgency, family environment, and child protection. In case of management necessity for those points, the professionals related to the mother and the child contacted with the other professionals and performed multidisciplinary supports [46, 48].

We also developed follow-up sheets (See Additional file 1) [46] to support women at risk of psychosocial problems. The follow-up sheets targeted case formulation. These sheets were used by the public health nurses. The follow-up sheet empowered public health nurses, who are non-psychiatric professionals, to assess mental health problems. When non-psychiatric professionals assess mental health problems, the assessments can differ depending on the experience and clinical skill level of the assessing professional [48, 46]. The assessment tool was used so that public health nurses could provide consistent assessment and follow-up with mothers at risk for psychosocial problems. Case formulation has been used by various professionals in the field of mental health cares, including psychologists, psychiatrists and social workers [56, 57]. Case formulation is an important component of intervention for suicide prevention (e.g. [1]). The contents of the follow-up sheets enabled case formulation [56, 57] and helped the nurses better understand the biopsychosocial aspects, of both the mother and child, systematically and informed them about care plan development.

We designed the interventions to not require structural or financial changes to the existing healthcare system, and sought to optimize the healthcare resources within the study region. With reference to the UK Medical Research Council recommendations for complex interventions [58], we closely monitored intervention intensity, described the narrative intervention process in detail, and investigated the levels of exposure to interventions in the post-intervention survey.

### Procedures

We assessed the mothers’ suicidal ideation using the Japanese version [59] of the EPDS [8]. The EPDS is a useful mental health screening instrument for the perinatal period [16], and has been used to assess the effects of interventions on women’s mental health [60]. It consists of 10 items, and each item is scored on a 4-point scale (0 to 3), with a minimum total score of 0 and a maximum of 30. The item 10 of the EPDS assesses thoughts of self-harm. Possible responses include 0 = never, 1 = hardly ever, 2 = sometimes, and 3 = quite often (i.e., the minimum and maximum scores of the self-harm question are 0 and 3, respectively). The self-harm question of the EPDS has been used for assessing suicidal ideation in the perinatal period [9, 61] and is considered valid [9]. We also assessed the mothers’ mental health using the total score of the EPDS. We assessed the mothers’ mental health at the 3- to 4-month infant medical examination (T1) and the 7- to 8-month infant medical examination (T2) using the total score of the EPDS, which means that all those outcomes were taken at the time points after the intervention of the intervention group for both the control and intervention groups. However, the baseline assessments using the EPDS were not taken in this study.

### Statistical analysis

To assess whether there were any significant differences in women’s ages between the intervention group and the control group, two sample t-tests were performed. To examine the effects of the intervention program for reducing maternal suicidal ideation, we employed a 2 × 2 repeated measures analyzes of variance using [Group] (intervention group or control group) as a between-subjects independent variable (IV) and the EPDS’s self-harm question score as a repeated measure IV for [Time] (T1 and T2).

1. The primary endpoint

The primary endpoint of this study was the simple main effect of [Group] on the score of the self-harm question of the EPDS at T1. This endpoint measured the effect of the intervention program on reducing suicidal ideation, and helped determine whether the intervention and the control groups had significantly different self-harm question scores at T1.

2) The secondary endpoints

This study had three secondary endpoints.

2.1) The simple main effect of [Group] on the total score of the EPDS at T1
2.2) The interaction of [Group] × [Time] on the score of the self-harm question of the EPDS
2.3) The interaction of [Group] × [Time] on the total score of the EPDS

2.1) was put for measuring the effect of the intervention program on maternal mental health improvements, and to investigate whether the intervention and the control groups had significant difference in the score of the self-harm question of the EPDS at T1. 2.2) and 2.3) measured the continuous effects of the intervention program, and measured if a significant between-group difference was evident at T1 and T2, to determine if the intervention program was effective, as we hypothesized.

3. Additional analyses

Besides those endpoints, we performed the following additional analyses:

3.1) The simple main effect of [group] on the self-harm question of the EPDS at T2
3.2) The simple main effect of [group] on the total score of the EPDS at T2

To consider intraclass correlations, we also performed the following linear mixed effect model analyses:

3.3) A multilevel analysis with the individual as the random effect and [group], [time] and the interaction of [group] × [time] on the self-harm question of the EPDS as fixed effects
3.4) A multilevel analysis with the individual as the random effect and [group], [time] and the interaction of [group] × [time] on the total score of the EPDS as fixed effects

Due to the odds frequency distribution of the primary endpoints (the self-harm question of the EPDS at Time 1), we performed ordered logistic regression analysis with the self-harm question of the EPDS as the ordered dependent variable and with group (the intervention group or the control group) as the independent variable. We tested the proportional odds assumption using the score test [62].

3.5) A sub-analysis considering the odds frequency distribution of the primary endpoints

Statistical significance was set at *p* < 0.05. All data analyzes were performed with the SPSS software program version 22.0 J for Windows (SPSS Inc., Tokyo, Japan).

## Results

The mean age of control group participants was 31.98 years (standard deviation [SD] = 4.85) and 31.82 years (SD = 5.18) for the intervention group. There was no statistically significant difference in mean age between the two groups (*p* = 0.755). Participant demographics of the two groups are shown in Table 1.

**Table 1:** Basic characteristics of the participants of the intervention and control groups

	Missing: total	Total	Missing	Intervention group (*n* = 230)	Missing	Control group (*n* = 234)
		Range [Min, Max]		Range [Min, Max]		Range [Min, Max]
Mother’s age	36	31.90 (5.01)	13	31.98 (4.85)	23	31.82 (5.18)
		[17, 49]		[17, 49]		[17, 43]
Father’s age	38	33.98 (5.79)	14	34.12 (5.99)	24	33.84 (5.61)
		[18, 54]		[18, 54]		[21, 54]
No sibling		236		117		119
First sibling						
number				113		115
Sex (male/female)			1	60/53	2	54/58
Age		4.34 (3.27)		4.77 (3.53)		3.90 (2.93)
		[0, 20]		[1, 20]		[0, 18]
Second sibling						
number			25	34	34	25
Sex (male/female)	59		25	18/16	34	11/14
Age		4.68 (3.19)		4.76 (3.53)		4.56 (2.71)
		[2, 13]		[2, 13]		[2, 11]
Third sibling						
number		6		3		3
Sex (male/female)		6/0		3/0		3/0
Age		6.00 (3.89)		6.60 (4.34)		5.00 (3.61)
		[3, 11]		[3, 11]		[3, 9]
Fourth sibling						
number		4	0	3	0	1
Sex(male/female)				2/1		0/1
Age		11.0 (5.00)		13.5 (7.50)		6.00 (0.00)
		[6, 21]		[6, 21]		[6]
Method of birth			4		3	
Spontaneous vaginal birth				181		195
Instrumental vaginal birth				6		5
Cesarean section				39		31
Delivery week		39.12 (1.35)		39.12 (1.33)		39.13 (1.38)
		[32, 42]				
Birth weight		2916.87 (737.59)		3046.56 (387.68)		2773.57 (971.11)
		[1745, 4148]		[1874, 4148]		[1745, 3890]

**Fig. 4:**
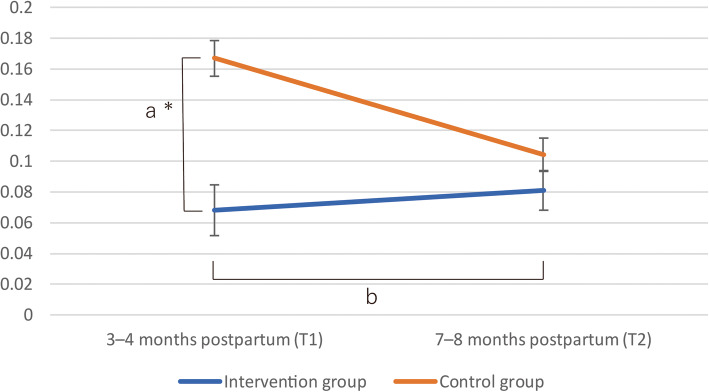
Results of the self-harm question of the Edinburgh Postnatal Depression Scale. Footnotes: The mean response to the self-harm question of the Edinburgh Postnatal Depression Scale (EPDS) (=the y-axis) was significantly lower compared with that of the control group (a: simple main effect *p* = 0.014). There was no significant effect in the two-way interaction between [Group] × [Time] on the self-harm question of the EPDS (b: *p* = 0.111). * = *p* < 0.05

**Fig. 5:**
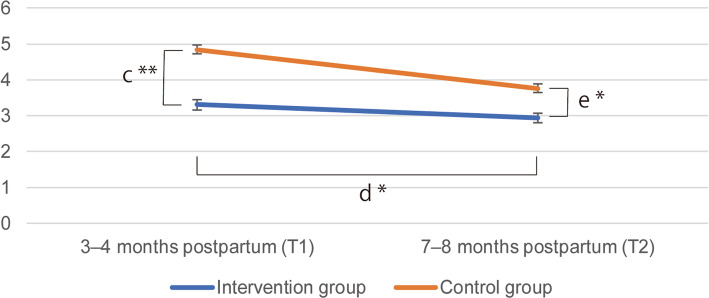
Results of the total score of the Edinburgh Postnatal Depression Scale. Footnotes: The mean total score of the Edinburgh Postnatal Depression Scale (EPDS) (= the y-axis) was significantly lower in the intervention, compared with the control group (c: simple main effect ; *p* < 0.001). There was a significant effect in the two-way interaction between [Group] × [Time] on the self-harm question of the EPDS (d: *p* = 0.049). There was significant main effect on the total score of the EPDS at T2 (e: *p* = 0.014). * = *p* < 0.05 and ** = *p* < 0.001, respectively

In 2015, there were 2017 births in Nagano city, and in 2016 there were 2952 births. There was an overlap period in April 2016 during which both the control and the intervention group participants existed. In that period, there were no women who were followed up by the public health nurses due to expressing suicidal ideation. The results of the primary and secondary endpoints are shown in Figs. 4 and 5.

The results of the primay and secondary endpoints The simple main effect of [Group] on the self-harm question of the EPDS at T1, which was the primary endpoint of this study, showed that the score of the intervention group was significantly less than that of the control group (F (1, 407) = 6.120, *p* = 0.014, η^2^ = 0.015). As for the secondary endpoints, the simple main effect of [Group] on the total score of the EPDS at T1 showed that the intervention group score was significantly less than the control group score (F (1, 407) = 17.034, *p* < 0.001, η^2^ = 0.037). There was not a significant [Group] × [Time] interaction, F (1, 407) = 2.546, *p* = 0.111, η^2^ = 0.006 with the self-harm question and time (T1 to T2), with the participants in the intervention group exhibiting a reduction in mean score, while the control group score increased. There was a significant [Group] × [Time] interaction, F (1, 407) = 3.914, *p* = 0.049, η^2^ = 0.009 with the total score of the EPDS over time (T1 to T2), with the participants in the control group exhibiting a significantly larger reduction in the mean total EPDS score.

The results of the additional analyses:

3.1) There was no significant main effect on the self-harm question of the EPDS at T2 (F (1, 462) = 0.47, *p* = 0.492, η^2^ = 0.001).
3.2) There was significant main effect on the total score of the EPDS at T2 (F (1, 460) = 6.06, *p* = 0.014, η^2^ = 0.013).
3.3) There was no significant [Group] × [Time] interaction on the self-harm question of the EPDS in the multilevel analysis (F[1448] = 1.69, *p* = 0.091)
3.4) There was a marginal significant [Group] × [Time] interaction on the total score of the EPDS in the multilevel analysis (F(1,451) = 1.95, *p* = 0.052).
3.5) The distribution of the answer of the self-harm question of the EPDS is shown in Additional file 2. The odds ratio derived from the ordered regression model was 2.982 [95% CI = 1.405 to 6.328] (*p* = 0.0044). The proportional odds assumption was verified by the score test (Chi-square = 0.755, degrees of freedom = 2, Pr > Chi-square = 0.686).

## Discussion

### Principal findings

The intervention program exhibited significantly decreased suicide ideation at 3–4 months postnatally. In the intervention group, mental health was improved at 3–4 months (T1) and 7–8 months postnatally (T2).

### Strengths and weaknesses

To the best of our knowledge, this study is the first to suggest the effectiveness of an intervention program for reducing suicidal ideation in postpartum women. This program has several strengths. First, it does not require any extra human resources or facilities and can be performed by adding maternal mental healthcare and suicidal ideation management to usual care practices for mothers and their children. Second, the program has a clear referral and management pathway for managing perinatal mental health, depended on the urgency of women’s psychiatric problems and childcare situation. The NICE guideline for antenatal and postnatal mental health suggests that clinical networks should be established for perinatal mental health services and managed by a coordinating board of healthcare professionals, commissioners, managers, service users, and careers [16]. This system comprises a specialist multidisciplinary local perinatal service, which provides direct services, consultation, and advice within the context of maternity services, other mental health services, and community series, as per NICE guidelines [16]. Regarding maternal suicide prevention, the assessment of the urgency of maternal mental health problems and child protection needs, which our referral and management pathway contained, is essential.

This study is associated with the following limitations. Firstly, we assessed suicidal ideation in the postnatal period using the EPDS and clinical interviews by public health nurses. No psychiatric professionals were used to assess suicidal ideation based on a structured interview. However, a previous study demonstrated the validity of the self-harm question of the EPDS for measuring suicidal ideation [9]. Thus, we think the results of the self-harm question could be regarded as valid for measuring participants’ suicidal ideation. Secondly, this study demonstrated program effectiveness for decreasing suicidal ideation in the population sample, but we did not determine whether the program decrease the number of postnatal suicides. Those at increased risk of suicide should receive timely intervention. Using a population-based intervention, the self-harm question of the EPDS could identify mothers at increased risk of suicide; therefore, a high-risk approach like that used with intervention program, may be an effective means of decreasing postnatal suicide. Thirdly, this study had a non-randomized controlled study design, which was the same as that used in our previous study [44]. This design means that other mechanism may explain the effects of the intervention observed in the present study. Further studies, including randomized controlled trials using the present intervention program, are needed. Fourthly, the present study lacked baseline assessments of EPDS scores before the intervention. However, the participants in the intervention and control groups were recruited at the same place (Nagano City), suggesting that the baseline scores of both groups were similar. Fifthly, the study design of the present study could include a contamination bias [63] regarding the intervention for suicidal ideation. Since no women who were followed up by the public health nurses due to expressing suicidal ideation within the overlap period of the control and the intervention group (i.e. April 2016), the suicide prevention intervention of the present program was not performed for the control group participants in that period. In addition, we think that an important mechanism of the effectiveness of the present intervention program was derived from the psychosocial risk assessment using the EPDS, including the suicidal ideation assessment. We do not think that the contamination bias had a significant effect on the results. Sixthly, the study design may have resulted in the inclusion of some seasonal confounding factors. Most of the participants in the control group delivered in June to October, while most of the participants in the intervention group delivered in November to January. There is no consensus about whether or not seasonality can affect peripartum depressive symptoms, with some studies suggesting that seasonality can indeed affect peripartum depressive symptoms [64–70], while others find no such association [71–73]. We therefore cannot suggest the degree to which the seasonality of the participants’ deliveries affected the results. Further investigations will be needed in studies with a design that allows for the exclusion of the potential confounding factor of seasonality.

### Comparison with other studies

For suicide prevention in general, it is important to assess the risk of suicidal ideation and then provide follow-up and support to women at increased risk of suicide using a high-risk approach [74]. This was also suggested by our previous study in Suzaka City, Japan [44]. In that study, public health nurses conducted psychosocial risk assessments for antenatal women and then performed intensive follow-up, if necessary. However, in this program, they did not assess suicide ideation but rather supported women at risk of psychosocial problems, though not from the viewpoint of suicide prevention. Their results showed that mental health of women in the intervention group was significantly improved compared with the control group, as indicated by the mean total score of the EPDS. However, the self-harm question of the EPDS, which is the indicator of the suicide ideation, was not significantly improved by the intervention program in Suzaka City. This differs from our current results, although both studies intervened using mental health supports for women in the postnatal period. We think this difference might be because the intervention group was specifically assessed for suicidal ideation and, if suicidal ideation was indicated, the mother received intensive support services. To prevent suicidal ideation, aggressive assessment and treatment should be included in standard community-based maternal care.

We think the effectiveness mechanism of the present intervention program partly derived from the same mechanism of our previous intervention program (the Suzaka trial) [44]. Our previous intervention program used the EPDS interviews conducted by the public health nurses, which played the following roles: 1) opening up the conversation about psychosocial issues; 2) raising awareness and educating pregnant women about the various psychiatric and psychosocial conditions; and 3) developing good relationships with the women by inquiring about their psychosocial problems. These allow the establishment of trust, open and frank information receiving, in turn, to a better understanding of the needs of the expectant women, which led to early intervention if they needed, and their better access to the maternal and child health services [44] .

In addition to the intervention system employed in the Suzaka trial, the present study’s intervention program had a characteristic approach that involved using case management intervention for women with suicidal ideations [75]. Case management is a comprehensive psychosocial care approach performed according to the patient’s individuality and has been shown to be effective in particularly complex cases. Continuum case managements based on psychiatric and psychosocial assessments has been shown to prevent recidivation of suicide attempts and self-harm [75, 76]. In Kawanishi et al.’s “Action-J”, which is a representative evidence-based suicide prevention program, their case management approach consists of the following four components: 1) psychological crisis intervention using appropriate communication methods; 2) accurate psychiatric and psychosocial assessments, including confirmation of the existence of suicidal ideation; 3) psychoeducation based on the results of 2); and 4) continuous case management focused on adherence to psychiatric treatment and considering each patient’s individuality. The follow-up system of our intervention program matched Action-J’s case management components. Compared to the four components of Action-J, our program has the following characteristics: 1) crisis intervention performed by public health nurses when deemed necessary (See Additional file 1: Judgement criteria of the timing of crisis intervention); 2) the comprehensive assessment of psychosocial risk by public health nurses using the EPDS and “follow-up sheet”, and if the nurses noted possible suicidal ideation with the women, they confirmed its existence; 3) psychoeducation performed by public health nurses to manage suicidal ideation and give advice on seeking help and using help resources for women with suicidal ideation; and 4) meetings by a multidisciplinary professional team to discuss how to support these women, with the team including public health nurses and mental health professionals (i.e. psychiatrists and psychologists) performing follow-up. Since Action-J’s case management system has evidence supporting the prevention of repeat suicide attempts and self-harm, we believe that the significant reduction in suicide ideation according to the EPDS in the intervention group compared to the control group may be due to the application of the case management system in this program.

On the other hand, the main interventionists in our program were public health nurses who also cared for the mothers and their children. Our results indicate the effectiveness of public health nursing supports for reducing maternal suicidal ideation and improving maternal mental health. In addition, in our intervention program, public health nurses and other related professionals can collaborate and administer material and child healthcare through a multidisciplinary clinical network. Our intervention program also provides public health nurses with a routine mental health screening system, and a systematic follow-up sheet for mental healthcare. Our findings suggest that the role of the public health nurse is pivotal for ensuring adequate and community-based maternal mental healthcare. If nurses perform integrated mental healthcare, like routine mental health screening, collaborate with other professionals, and follow-up with mothers and children using systematic assessments and care plans, their maternal mental health interventions will likely be very effective.

The results of the main and sensitivity analysis for the self-harm question of the EPDS and the total score of the EPDS are considered to be consistent. Both the sensitivity analyses’ estimate values and their standard errors were nearly the same as those of the RMANOVA. The finding of 1) (the simple main effect of [Group] on the score of self-harm question of the EPDS at T1(significant) and 3.1) (the same simple main effect at T2: not significant) and 2.2 (the interaction of [Group] × [Time] on the score of the self-harm question of the EPDS: not significant) suggested that this intervention program can be effective at 3–4 months postpartum, but the effectiveness was not observed to continue at 7-8 months postpartum. A Japanese study reported that the number of women who committed suicide from 7 to 8 months after delivery was low compared to that from 3 to 4 months after delivery, although suicides among women were reported throughout the first year postpartum [77]. These results may be because the period of 3–4 months after delivery is the highest suicide risk period in the first 12 months postpartum.

Regarding women’s general mental health (as measured by the total score of the EPDS), the results suggested that this intervention program might be effective at both 3–4 and 7–8 months after delivery. Since the prevalence of postnatal depressive episodes is still slightly high at 7–8 months postpartum [78–81], the results may indicate the effectiveness of the intervention program on the intervention group in the period. The results of the linear mixed model analyses indicated that, if the intraclass correlations are considered, the estimated values and their standard errors were nearly equal to those of the ANOVA analyses (2.2 and 2.3) and those results are thus considered to be consistent.

The distribution of the answers of the self-harm question of the EPDS, for the intervention and the control groups, indicate this intervention approach may be effective for mothers with low to moderate suicidal ideation. The frequency of “hardly ever” and “sometimes” as response choices to the self-harm question of the EPDS were low in the intervention group, compared with the control group. However, the frequency of the answer “yes, quite often” was the same between the two groups. Since the frequency of response “yes, quite often” in the self-harm question of the EPDS is very rare, this study lacks evidence to infer the effects of the program on those who have severe suicidal ideation. However, gatekeeper functions, including early detection and appropriate referrals to psychiatric services, are effective for both suicide prevention [7, 53] and perinatal mental health care [44]. This intervention program had clear referral and management protocol for women with severe suicidal ideation, by way of the related professionals who served both mother and child. Further research is needed to apply this intervention program to a larger study cohort, and for qualitative research to examine its effects on those with severe suicidal ideation.

### Meaning of the study

Postnatal suicide prevention should be routinely performed in the context of postnatal care, not as an independent suicide prevention program. Our postnatal suicide prevention program consists of i) suicidal ideation screening using the EPDS, ii) assessment and follow-up based on mental health formulations, iii) management and care based on a clinical pathway. It will be difficult to devote new staff and additional budget for postnatal prevention, in addition to existing community-based usual postnatal care practices. However, i) to iii) can be performed in the context of ordinary postnatal care by maternal and child care professionals. As our results showed, the mental healthcare package can improve maternal mental health as well as suicide ideation. We believe that a postnatal mental health package that includes suicide ideation assessments and a case management approach, like this program, should be applied to general postnatal care.

### Unanswered questions and future research

Although our results suggest that the intervention program reduced suicidal ideation in postnatal women, it is unknown whether the program could decrease the number of actual postnatal suicides. Since postnatal suicide is fortunately rare (50–60 per 100,000 deliveries in Japan), a larger-scale population-based approach is needed to investigate the effectiveness of this intervention program on decreasing the number of postnatal suicides.

## Conclusions

The current study proposes an integrated community mental health care program aimed at preventing suicide in women at risk of psychosocial problems during the postnatal period and suggested its effectiveness for reducing suicidal ideation at 3–4 months and improving maternal mental health at 3–4 and 7–8 months postnatally.

## Supplementary information

**Additional file 1.** Formulation and follow-up sheet (translated into English).

**Additional file 2.** Distribution of the answers of the item 10 (self-harm question) of the Edinburgh Postnatal Depression Scale (EPDS) at 3–4 months postpartum (T1).

**Additional file 3.** Suicide intervention criteria.

**Additional file 4.** CONSORT 2010 checklist.

## Data Availability

The datasets generated and/or analyzed during the current study are not publicly available because of Nagano city’s privacy policy.

## Abbreviations

EPDS: Edinburgh Postnatal Depression Scale
NICE: National Institute for Health and Care Excellence
