# Supplementary material for: An integrated community mental healthcare program to reduce suicidal ideation and improve maternal mental health during the postnatal period: the findings from the Nagano trial
Source: BMC Psychiatry. 2020 Jul 29;20:389. doi: 10.1186/s12888-020-02765-z (PMC7390164; doi:10.1186/s12888-020-02765-z)
Supplement: Supplementary file 1 — Additional file 1. Formulation and follow-up sheet (translated into English). [file 12888_2020_2765_MOESM1_ESM.pdf]

Additional file 1. Formulation and follow-up sheet (translated into English)

Support Sheet for Mother and Child

Date / /

By

Mother's name Child's name

Family environment

Mother's physical condition

☐ Wound aching ☐ breastfeeding problems ☐ Others

Mother's psychological problems

☐ Depression ☐ Anxiety ☐ Cannot seek help when needed

EPDS total score ( )

Other psychosocial problems which were detected in the Mother Infant Bonding Scale and the Checklist for Supporting Mother and Child

Observed findings relating to mother and child

Childcare findings

☐ Attachment ☐ Child care skill

Concerning the mother

Concerning the child

Usable support resources

Support plan
