# Supplementary figures and images for: An integrated community mental healthcare program to reduce suicidal ideation and improve maternal mental health during the postnatal period: the findings from the Nagano trial
Source: BMC Psychiatry. 2020 Jul 29;20:389. doi: 10.1186/s12888-020-02765-z (PMC7390164; doi:10.1186/s12888-020-02765-z)

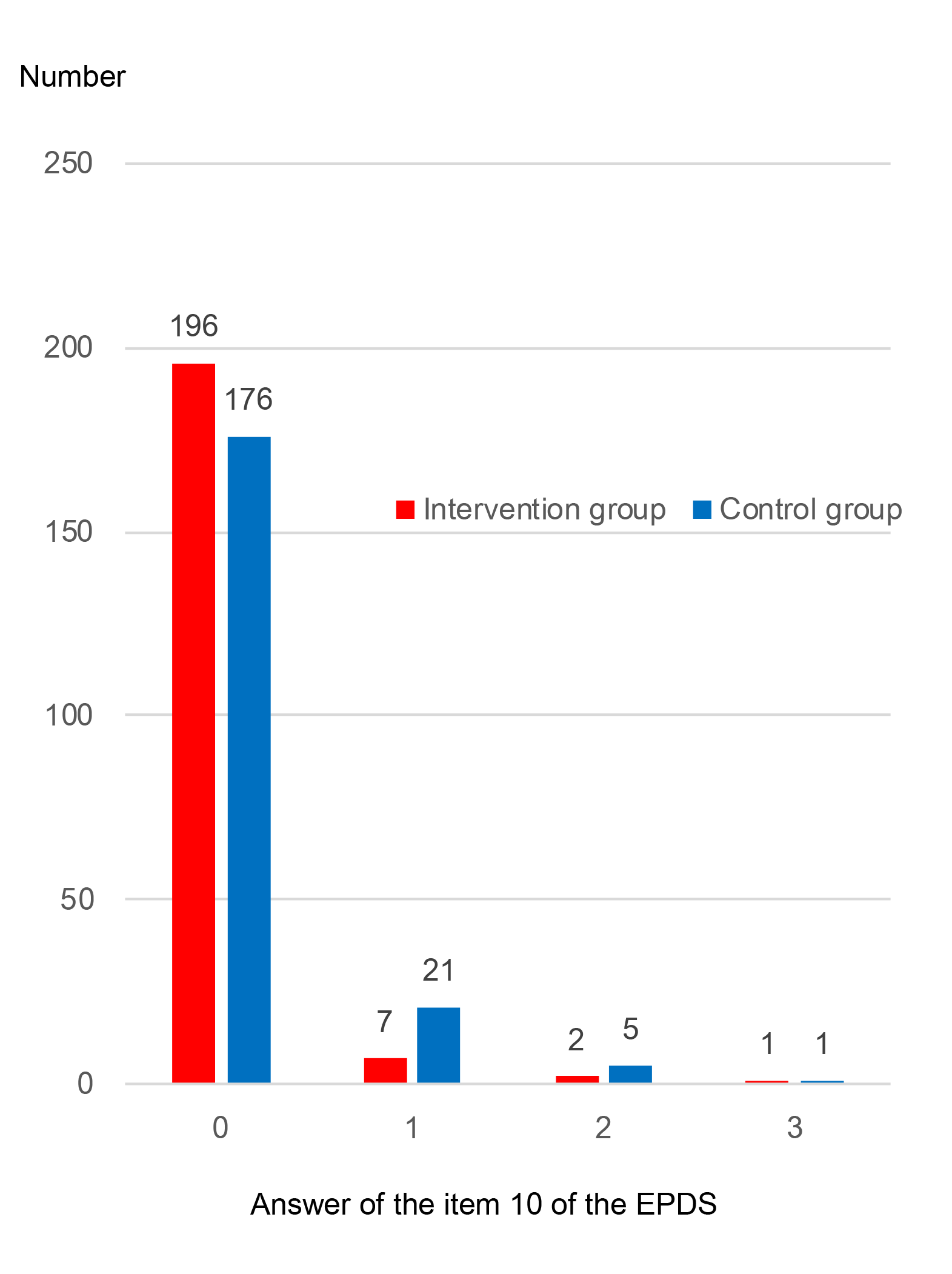

Supplement: Supplementary file 2 — Additional file 2. Distribution of the answers of the item 10 (self-harm question) of the Edinburgh Postnatal Depression Scale (EPDS) at 3–4 months postpartum (T1). [file 12888_2020_2765_MOESM2_ESM.tif]
