# Supplementary material for: An integrated community mental healthcare program to reduce suicidal ideation and improve maternal mental health during the postnatal period: the findings from the Nagano trial
Source: BMC Psychiatry. 2020 Jul 29;20:389. doi: 10.1186/s12888-020-02765-z (PMC7390164; doi:10.1186/s12888-020-02765-z)
Supplement: Supplementary file 3 — Additional file 3. Suicide intervention criteria. [file 12888_2020_2765_MOESM3_ESM.pdf]

Additional file 3. Suicide intervention criteria

The following conditions should be regarded as "emergent conditions that merit immediate intervention":

1. The woman has suicidal ideation that she cannot control on her own.
2. The psychiatric symptoms (i.e. hallucination and delusion) suddenly occur or worsen.
3. The woman is at risk of harming herself or others.
